# Supplementary material for: Specific Subtypes of Carcinoma-Associated Fibroblasts Are Correlated with Worse Survival in Resectable Pancreatic Ductal Adenocarcinoma
Source: Cancers (Basel). 2023 Mar 30;15(7):2049. doi: 10.3390/cancers15072049 (PMC10093167; doi:10.3390/cancers15072049)
Supplement: Supplementary file 1 [file cancers-15-02049-s001.zip › cancers-2271442-supplementary.pdf]

Supplement Information

Supplementary Figure S1. Representative images of the performed immunohistochemical stainings (left: low, right: high): (**A**) FAP, (**B**) PDGFR beta, (**C**) Periostin, and (**D**) SMA. Scale bar: 50 μm

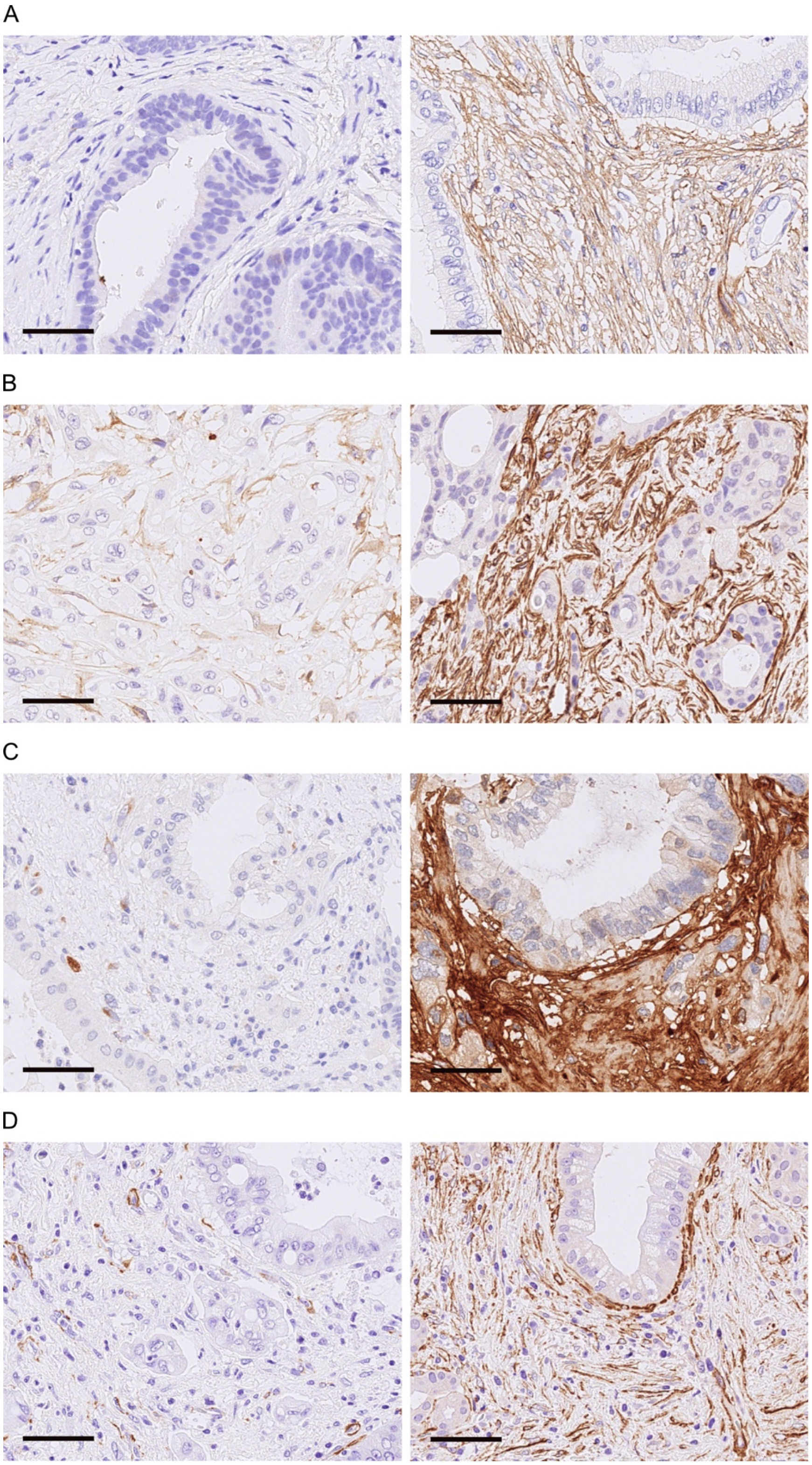

Supplementary Table S1. Detailed antibody information. A: appendix vermiformis, w/o: without pretreatment.

Antibody	Manufacturer	Clone	Dilution	Pretreatment	Control tissue	Positive control	Negative control	Order number
SMA	Dako/ CE	1A4, mouse	1:4000	w/o	A	Smooth muscle cells	Epithelial cells	M0851
FAP	Abcam	EPR20021	1:200	Citrat	Colon, Mamma	Stromal fibroblasts	Cancer cells	Ab207178
PDGFR beta	Abcam/ RUO	Y92, rabbit	1:300	EDTA	Prostate	Stromal cells	Cancer cells	Ab32570
Periostin	Abcam	EPR19934	1:2000	EDTA	Colon	Extracellular matrix	Epithelial cells	Ab219056

Supplementary Table S2. Univariate cox regression. Bold print marks p-values below 0.05.

Characteristic	Borders	Hazard Ratio	95 % confidence interval	p - value
**Sex**	female vs male	0.894	0.679 - 1.177	0.425
**Age**	^{3} 65 vs < 65	0.888	0.667 - 1.183	0.418
**Preoperative staging**				0.052
	borderline vs primarily resectable	1.509	1.024 - 2.226	**0.038**
	Locally advanced vs primarily resectable	3.780	0.525 - 27.208	0.187
**Neoadjuvant therapy**	yes vs no	1.296	0.826 - 2.034	0.260
**pT**				**0.004**
	2 vs 1	1.525	0.783 - 2.970	0.214
	3 vs 1	2.342	1.226 - 4.475	**0.010**
	4 vs 1	2.872	0.979 - 8.431	0.055
**pN**	1 vs 0	2.123	1.521 - 2.963	**< 0.001**
**R**	^{3} 1 vs 0	1.573	1.203 - 2.056	**< 0.001**
**Pn**	1 vs 0	1.432	1.013 - 2.025	**0.042**
**L**	1 vs 0	1.210	0.909 - 1.612	0.192
**V**	1 vs 0	1.370	1.013 - 1.854	**0.041**
**SMA**	high vs low	1.357	1.025 - 1.795	**0.033**
**FAP**	high vs low	1.190	0.904 - 1.567	0.216
**PDGFR**	high vs low	1.177	0.894 - 1.550	0.246
**Periostin**	high vs low	1.155	0.878 - 1.521	0.304
